# Pan-genome survey of *Septoria pistaciarum*, causal agent of Septoria leaf spot of pistachios, across three Aegean sub-regions of Greece

**DOI:** 10.3389/fmicb.2024.1396760

**Published:** 2024-06-11

**Authors:** Antonios Zambounis, Anastasia Boutsika, Naomi Gray, Mohitul Hossain, Michael Chatzidimopoulos, Dimitrios I. Tsitsigiannis, Epaminondas Paplomatas, James Hane

**Affiliations:** ^1^Hellenic Agricultural Organization - DIMITRA (ELGO - DIMITRA), Institute of Plant Breeding and Genetic Resources, Thessaloniki, Greece; ^2^Centre for Crop and Disease Management, Department of Molecular and Life Sciences, Curtin University, Perth, WA, Australia; ^3^Laboratory of Plant Pathology, Department of Agriculture, International Hellenic University, Thessaloniki, Greece; ^4^Laboratory of Plant Pathology, Department of Crop Science, Agricultural University of Athens, Athens, Greece

**Keywords:** *Septoria pistaciarum*, pistachio, Mycosphaerellaceae, plant-pathogen, pathogenicity effectors

## Abstract

*Septoria pistaciarum,* a causal agent of Septoria leaf spot disease of pistachio, is a fungal pathogen that causes substantial losses in the cultivation, worldwide. This study describes the first pan-genome-based survey of this phytopathogen—comprising a total of 27 isolates, with 9 isolates each from 3 regional units of Greece (Pieria, Larissa and Fthiotida). The reference isolate (SPF8) assembled into a total of 43.1 Mb, with 38.6% contained within AT-rich regions of approximately 37.5% G:C. The genomes of the 27 isolates exhibited on average 42% gene-coding and 20% repetitive regions. The genomes of isolates from the southern Fthiotida region appeared to more diverged from each other than the other regions based on SNP-derived trees, and also contained isolates similar to both the Pieria and Larissa regions. In contrast, isolates of the Pieria and Larissa were less diverse and distinct from one another. Asexual reproduction appeared to be typical, with no MAT1-2 locus detected in any isolate. Genome-based prediction of infection mode indicated hemibiotrophic and saprotrophic adaptations, consistent with its long latent phase. Gene prediction and orthology clustering generated a pan-genome-wide gene set of 21,174 loci. A total of 59 ortholog groups were predicted to contain candidate effector proteins, with 36 (61%) of these either having homologs to known effectors from other species or could be assigned predicted functions from matches to conserved domains. Overall, effector prediction suggests that *S. pistaciarum* employs a combination of defensive effectors with roles in suppression of host defenses, and offensive effectors with a range of cytotoxic activities. Some effector-like ortholog groups presented as divergent versions of the same protein, suggesting region-specific adaptations may have occurred. These findings provide insights and future research directions in uncovering the pathogenesis and population dynamics of *S. pistaciarum* toward the efficient management of Septoria leaf spot of pistachio.

## 1 Introduction

Pistachio (*Pistacia vera* L.) is an important crop in various regions around the world including the Mediterranean basin (Drais et al., 2023). The global economic value of pistachios is estimated to be over $1 billion annually, with the top five producers being the United States, Iran, Turkey, Syria and Greece (Mateos et al., 2022). *Septoria pistaciarum*—the causal agent of Septoria blight of pistachios, is an important pathogen reported across all pistachio-growing regions, including: the United States (Young and Michailides, 1989), Middle-East (Akgul et al., 2011), Central Asia (Ahmad et al., 2011) and the Mediterranean (Eskalen et al., 2001; Gusella et al., 2021; Lopez-Moral et al., 2022; Drais et al., 2023).

*Septoria pistaciarum* infects leaves, shoots, and nuts of pistachio trees, causing a variety of symptoms, including leaf spots, shoot blight, and nut rot (Crous et al., 2013) and can significantly reduce pistachio quality and yield (Drais et al., 2023). In leaves the symptoms of the disease appear on both sides from spring until the end of summer, where the pathogen produces conidia from pycnidial conidiomata that dispersed initiating new infections (Gusella et al., 2021). Pistachio trees may prematurely defoliate due to severe inoculum pressure and favorable environmental conditions (Drais et al., 2023). In turn, the bearing shoots may be also affected along with the he physiological processes of assimilation of carbohydrates necessary for bud differentiation (Drais et al., 2023). The host range of *S. pistaciarum* appears to be limited to pistachio trees, but the pathogen can survive in infected plant debris on the ground, in fallen leaves, or infected nursery stock (Chitzandis, 1956). Although other closely related species of the Mycosphaerellaceae also infect pistachio, *S. pistaciarum* is morphologically distinctive with angular leafspots confined by leaf-veins (Crous et al., 2013). Spread can occur by wind-blown spores which can disperse in long distances (Gusella et al., 2021), or by contact with infected plant material (Chitzandis, 1956).

As various aspects of the pathogen life cycle and epidemiology are still unclear, the management of this pathogen with commercial fungicides is quite a challenging task. Early studies report the good efficacy of copper and triazole fungicides against Septoria leaf spot of pistachios in USA (Call and Matheron, 1994) and Turkey (Çat, 2022). In Greece the standard farming practice includes the application of various copper formulations until flowering and then a combination of fungicides from the classes of demethylation inhibitors (DMIs), quinone outside inhibitors (QoIs), fourth generation succinate dehydrogenase inhibitors (SDHIs) and anilinopyrimidines (APs) at 14-day intervals for at least 2 months. Detecting latent infections within the pistachio tissues could be a crucial step in limiting the number of field sprays, as well as in ensuring a sustainable use of effective fungicides through an accurate spray program (Gusella et al., 2021).

Genomics has been extensively applied to some plant-pathogenic fungal taxa (Aylward et al., 2017), and has been a useful tool enabling bioinformatic inference and molecular biology methods to validate their pathogenicity mechanisms (Jones et al., 2018). In this context, *S. pistaciarum* has not yet received much attention, although it is related to other hemibiotrophic pathogens with comparatively extensive pan-genomic resources, including: *Zymoseptoria tritici* (Testa et al., 2015); *Pseudocercospora fijiensis* (Arango Isaza et al., 2016); *Ramularia collo-cygni* (McGrann et al., 2016; Stam et al., 2018; Sjokvist et al., 2019) and; *Cercospora* spp. (Chand et al., 2015; Orner et al., 2015; Albu et al., 2017; Vaghefi et al., 2017; Wingfield et al., 2017; Zeng et al., 2017a,b; Sautua et al., 2019; Gu et al., 2020; Lin et al., 2022; Cheng et al., 2023; Yang et al., 2023). A previous phylogenetic study based on 5 conserved loci—which distinguished *S. pistaciarum* from other pistachio-infecting *Mycosphaerellaceae* spp. (Crous et al., 2013)—amounts to the current extent of genomic study of this organism. This study represents the first analysis of the pan-genome of a local Mediterranean population of *S. pistaciarum*. These resources will enable the study of the genomic features driving the pathogenicity and biological complexity of this foliar pathogen, with new insights into its population dynamics and pan-genomic structure.

## 2 Methods

### 2.1 Origin of *Septoria pistaciarum* isolates and DNA extraction

**Figure 1:**
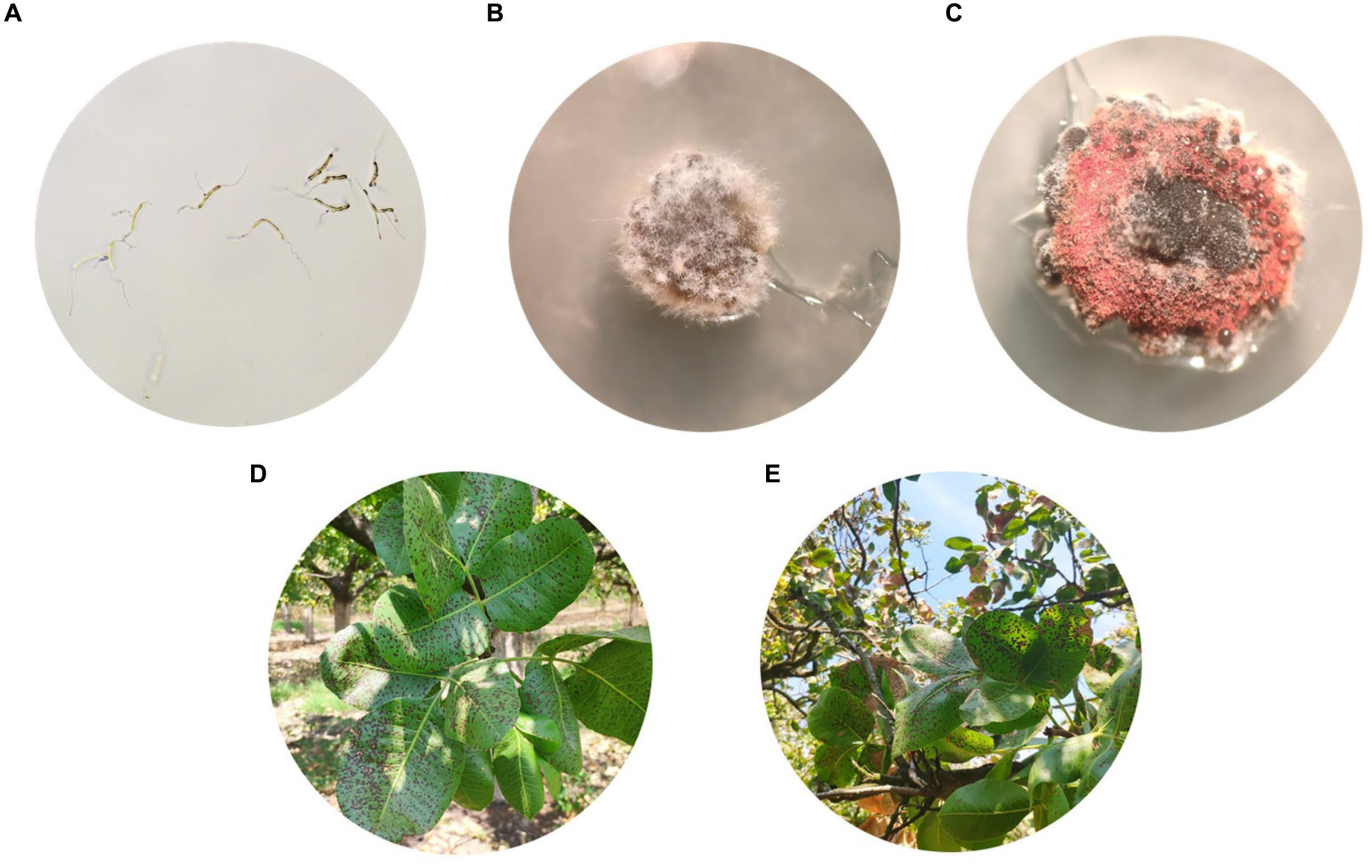
Morphological features of *Septoria pistaciarum*: **(A)** Germinated single-spore conidia on PDA after 48 h of incubation at 24°C in the dark. **(B,C)** Representative isolates SPF8 **(B)** and SPF9 **(C)** with grown on PDA after 4 weeks of incubation at 24°C in the dark; **(D,E)** Symptoms of Septoria leaf spot observed in pistachio leaves infected by SPF8 **(D)** and SPF9 **(E)**.

During the 2022 growing period, *S. pistaciarum* isolates were obtained in June from pistachio leaves showing leaf spot symptoms (cv. Aeginis) across three distinct sites located in the areas of Amfikleia, Fthiotida (38.629963 N, 22.681427E; code FTH), Kiparissia, Larissa (39.5138684 N, 22.5733432E; code LAR) and Kitros, Pieria (40.3785945 N, 22.607257E; code PIE). The trees were over 20 years old and trained under the standard open vase system to an average height of 10 m. A standard fungicide program based on the fungicides boscalid + pyraclostrobin (Signum 26,7/6,7 WG; BASF), fluxapyroxad (Sercadis 30 SC; BASF), pyrimethanil (Scala 40 SC; BASF), dodine (Syllit 544 SC; UPL), and copper (Cuprofix Ultra 40 WG; ADAMA) was applied on all orchards in spring. Each diseased leaf sample was transferred to the lab in a separate moist polyethylene bag to prevent cross-contamination and stored at room temperature for 72 h to induce the formation of cirrhi in the pycnidia. From each sample a single-spore isolate was obtained by slight touching a flamed wire loop onto a freshly formatted cirrhus of conidia from one spot per leaf randomly picked up. A sparse pycnidiospore suspension in 0.5 mL distilled water was prepared and spread onto Potato Dextrose Agar (PDA) in Petri dishes amended with 100 mg/L of streptomycin sulfate. After 48 h of incubation at 24°C in the dark, individual germinated single-spore conidia were transferred in glass test tubes with potato dextrose agar and stored until use (Figure 1). Pycnidiospores were hyaline, curved, with obtuse ends, having 1 to 5 septa (Chitzandis, 1956) typically of *S. pistaciarum*. In total, 27 isolates were obtained (with nine isolates representing each region; Table 1) and they were maintained in PDA tubes at 4°C. Because of the very slow growth of the hyphae, in order to obtain larger colonies in plates for further analysis each representative plate was seeded with 0.4 mL of a conidial suspension from each isolate (Dhingra and Sinclair, 1985). After 4 weeks of incubation in the dark, fungal DNA was extracted from conidiomata (Figure 1) of all isolates using the Quick-DNA™ Fungal/Bacterial Miniprep Kit (Zymo Research).

**Table 1:** Summary of genome assembly metrics for 27 isolates of *Septoria pisticiarum* isolated across 3 sub-regions of Greece (FTH, Fthiotida; LAR, Larisa; PIE, Pieria).

Genome assembly metrics (pan-genome)									
Isolate	REGION	Total (bp)	#seq	L50	N50	%repeat	%gene	mtDNA (bp)	#genes
SPF8*	**FTH**	**43,103,949**	**13,114**	**82**	**83,514**	**18%**	**35%**	**68,083**	**12,060**
SPF1	FTH	35,634,496	14,626	87	81,780	21%	42%	65,619	10,231
SPF2	FTH	36,861,711	17,851	95	72,148	21%	42%	67,037	11,211
SPF3	FTH	35,614,614	13,213	66	89,307	22%	42%	74,010	10,240
SPF4	FTH	34,516,584	14,917	94	75,883	20%	43%	67,145	10,065
SPF6#	FTH	36,233,712	15,620	85	82,249	21%	42%	62,438	10,435
SPF7	FTH	35,954,154	14,297	96	77,528	21%	42%	65,507	10,460
SPF9	FTH	34,980,395	14,049	80	86,643	20%	43%	65,689	10,255
SPF10	FTH	36,976,791	17,258	87	80,922	21%	41%	66,900	10,856
SPL11	LAR	37,450,640	19,530	97	72,604	19%	42%	66,904	11,714
SPL12	LAR	34,363,077	14,165	70	85,640	20%	43%	66,998	10,076
SPL13	LAR	39,014,206	17,059	127	64,390	18%	43%	66,935	12,857
SPL14	LAR	42,485,921	24,278	130	52,754	18%	42%	67,208	14,844
SPL15	LAR	35,883,402	15,976	100	70,740	21%	42%	67,116	10,425
SPL16	LAR	35,552,267	15,568	96	76,241	21%	42%	66,914	10,173
SPL18	LAR	36,603,906	16,877	101	70,970	21%	42%	66,916	10,800
SPL19	LAR	35,427,260	15,600	103	73,818	21%	42%	65,923	10,321
SPL20	LAR	35,679,390	13,677	91	99,182	22%	42%	76,841	10,313
SPP21	PIE	35,211,260	14,089	78	86,793	20%	43%	65,423	10,319
SPP22	PIE	35,541,349	13,729	69	97,572	21%	42%	66,580	10,277
SPP23	PIE	36,173,688	16,651	86	80,280	21%	42%	65,396	10,495
SPP24	PIE	35,051,173	13,699	73	96,192	20%	43%	66,394	10,133
SPP25	PIE	35,142,955	14,692	77	89,955	20%	43%	66,561	10,225
SPP26#	PIE	87,505,418	29,758	268	77,788	9%	31%	177,400	23,067
SPP27	PIE	35,105,626	14,095	75	82,083	20%	43%	68,003	10,356
SPP28	PIE	35,386,077	15,477	77	85,048	21%	42%	66,953	10,205
SPP30	PIE	36,554,020	16,975	85	79,773	20%	42%	65,592	10,965

^*^Reference isolate; ^#^Contamination detected.

### 2.2 Genome assembly

Fungal DNA from all isolates was randomly fragmented into 350 bp inserts, genomic libraries were constructed using the Novogene NGS DNA Library Prep Set (Cat No.PT004) and sequenced by a PE150 sequencing strategy. Illumina reads were trimmed by cutadapt v3.7 (-a AAGTCGGAGGCCAAGCGGTCTTAGGAAGACAA-A AAGTCGGATCGTAGCCATGTCGTTCTGTGAGCCAAGGAGTTG --minimum-length = 50 -- max-*n* = 3 -n2 -q30; Martin, 2011). All isolates were assembled by SPAdes v3.15.4 (--only_assembler –cov-cutoff auto; Bankevich et al., 2012). Assembly quality and gene set completeness was predicted via BUSCO v5.4.6 (Simao et al., 2015). Initial genome assembly metrics were assessed for contiguity and BUSCO completeness (Supplementary Data Sheet 1), from which the “SPF8” isolate was selected as a reference isolate. Reads for isolate ‘SPF8’ were merged and gap-filled with BBMerge (Bushnell et al., 2017) then *de novo* assembled with SPAdes as above. Genome assemblies of all isolates were further scaffolded were possible using pairwise comparisons vs. other isolates with ragtag v2.1.0 (scaffold: –remove-small -f 75 -r, merge; Alonge et al., 2022). Mitochondrial genomic DNA (mtDNA) was assembled via MitoZ v3.6 (Meng et al., 2019), and assembled mtDNA contigs were appended to the genome assemblies, while previously-assembled sequences matching mtDNA contigs were removed. Contamination checks for each assembly was performed via BLAST v2.12.0 (blastn -max_target_seqs 5 -evalue 1e-100 -perc_identity 95; Altschul et al., 1990) to the UNITE database (Abarenkov et al., 2010). Overall G:C content and AT-rich compartments were predicted with OcculterCut v1.1 using the reference isolate SPF8 genome assembly (Testa et al., 2016).

### 2.3 Prediction of genomic repeats, genes and functions

Repetitive sequences were predicted using Dfam TE Tools 1.88 (Lerat et al., 2016). Gene prediction was performed across all isolates in 2 rounds. In the first round, funannotate v1.8.15 (predict; max_intronlen 1000; Palmer and Stajich, 2020) was used to predict genes for all isolates. In order to provide further evidence for the accurate support of gene loci and their exon boundaries, transcriptomic data was obtained for the isolate SPF8. Thus, total mRNA was extracted in triplicate from the mycelial phase of this isolate grown on PDA medium from a fresh two-weeks-old culture. The Quick-RNA™ Fungal/Bacterial Miniprep kit (Zymo Research) was employed, and RNAseq reads were generated through Illumina platform (PE150 Novaseq 6000) using the Novogene NGS RNA Library Prep Set (PT042). RNAseq alignment vs. the reference isolate was performed with HiSAT2 v2.2.1 (max-intronlen 5000; dta; Kim et al., 2019), converted to GFF via Stringtie v2.2.1 (Levy Karin et al., 2020) and provided as input to funnannotate. Funannotate-predicted proteomes were clustered into orthogroups (including singletons) with ProteinOrtho v6.3.1 (selfblast; singles; Lechner et al., 2011). A representative pan-genome proteome dataset was selected from the longest member of each orthogroup, and used as input to MetaEuk Release 6-a5d39d9 (--easy-predict –max-intron 500; Levy Karin et al., 2020) for a second round of gene prediction in all isolates, and orthogroups with MetaEuk matches were retained as a representative ‘pan-genome’ proteome set. Functional annotations were predicted across the pan-genome proteome dataset with InterProScan v5.63-95.0 (Jones et al., 2014). Secretion and effector-like properties were predicted using Predector v1.2.7 (Jones et al., 2021). Trophic niche (i.e., biotrophy, necrotrophy, etc.) was predicted via CATAStrophy v0.1.0 (Hane et al., 2020; using HMMER 3.3) vs. dbCAN v10. Mating type genes were screened vs. a representative dataset (Wilken et al., 2017) using MetaEuk (as above). Secondary metabolite synthesis gene clusters were predicted in the reference isolate assembly (SPF8) with antiSMASH v6.1.1 (Blin et al., 2021). Fungicide-resistance mutations were predicted with the fungicide-resistance allele screening tool (FRAST) (Oliver et al., 2024).

### 2.4 Comparative genomics

Gene ortholog group presence-absence variation (PAV) across the *S. pistaciarum* pan-genome was determined based on matches to a pan-genome-wide representative proteome dataset using MetaEuk (see above). Genome sequencing reads were aligned to the SPF8 assembly with BWA v0.7.17-r1198-dirty (Li and Durbin, 2009). Genome sequencing reads were processed fastqToSam to generate raw ubam inputs, and passed through MarkIlluminaAdapters, MarkDuplicates, and combined with BWA alignmed bam data with MergeBamAlignment (-CREATE_INDEX true -ADD_MATE_CIGAR true) to generate alignmed uban inputs for variant calling. Variant calling was performed with GATK v4.2.6.1 (HaplotypeCaller -ERC GVCF –minimum-mapping-quality 20 –min-base-quality-score 20 -G StandardAnnotation -G AS_StandardAnnotation -G StandardHCAnnotation; McKenna et al., 2010). Bi-allellic single nucleotide polymorphisms (SNPs) were filtered and randomly selected for 1 per 5 kb with BCFTools v1.15 (+prune -w 5,000 bp -n1 -N rand; Danecek et al., 2021), and used to generate an unrooted distance-based tree via IQTree v2.2.2.7 (1,000 iterations, -bb 1000 -alrt 1000; Minh et al., 2020). The tree was visualized alongside selected and summarized predicted protein function data (mating types, effector-like proteins) with iTOL (mid-point root; Letunic and Bork, 2021).

## 3 Results and discussion

### 3.1 Assessment of genome quality and features

**Figure 2:**
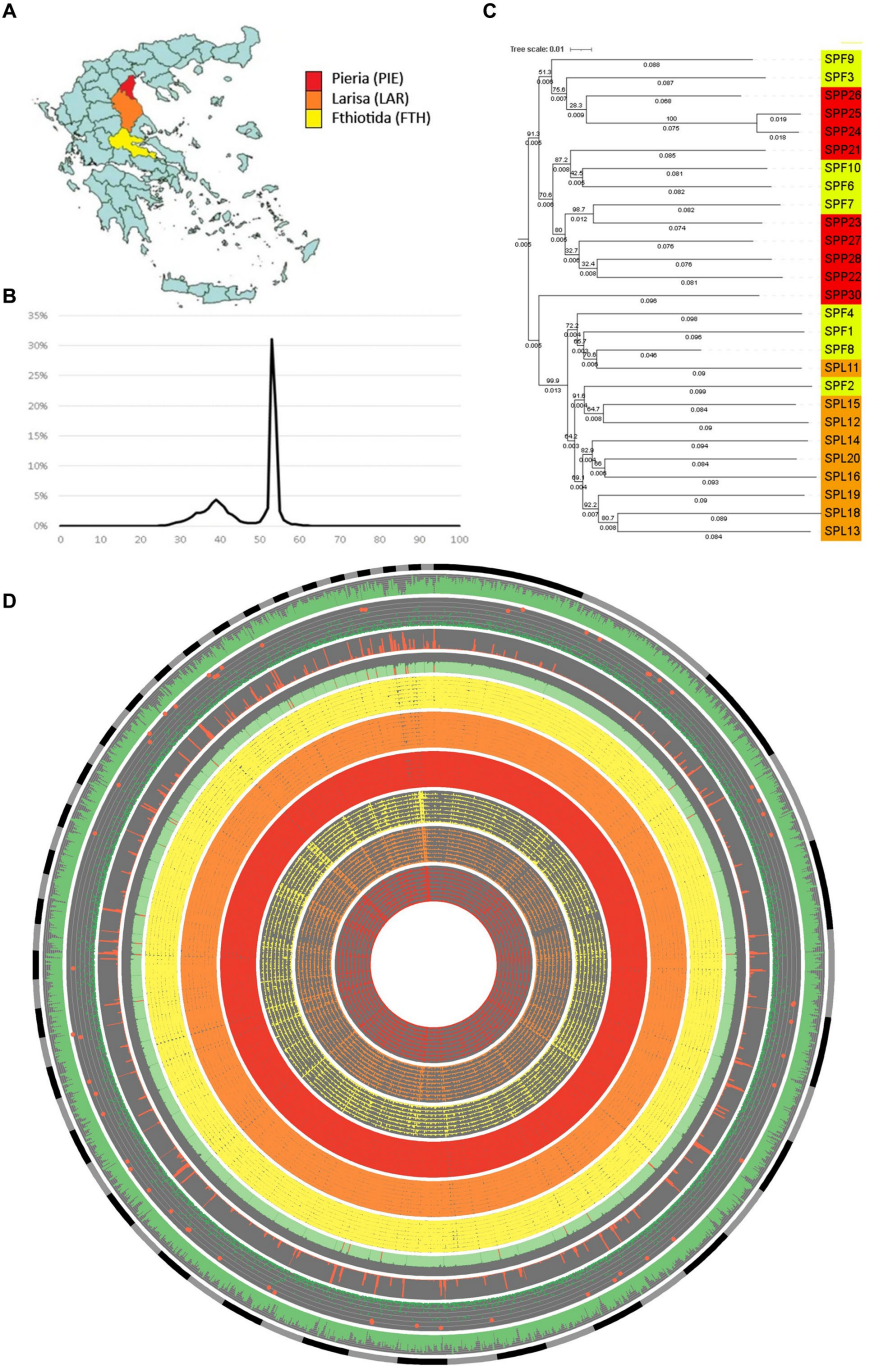
Summary of 27 *Septoria pistaciarum* isolates sampled across Greece and their genomic features. **(A)** Nine isolates each were sampled from 3 regions bordering the Aegean Sea: Pieria (PIE-red); Larisa (LAR-orange); Fthiotida (FTH-yellow). **(B)** G:C content (x-axis) summarized by the relative proportion of the reference isolate (SPF8) genome assembly (y-axis) indicated bi-modal G:C distribution with a significant proportion of the genome organized into AT-rich compartments **(C)** Midpoint-rooted distance-based tree of the 27 isolates based on SNP variant calling relative to the reference isolate. **(D)** A circular plot of genomic features relative to the reference isolate assembly (excluding sequences < 100 kb), with co-centric rings representing (in inward order): sequence length (gray and black); gene density (% coverage/10 kb, green); Predector effector-likelihood scores (−3 to 3, green, with scores >2 highlighted red); repeat density (% coverage/10 kb, red); G:C content (%G:C/10 kb, green, with <40% highlighted red); Homology to alternate isolates (%match coverage/10 kb, FTH = yellow, LAR = orange, PIE = red); Mutation density (from 1 to 500 SNPs/10 kb, FTH = yellow, LAR = orange, PIE = red).

The reference isolate (SPF8) assembled into a total of 43.1 Mb. All isolate assemblies exhibited ~70-120X sequencing depth (ex. SPP26), an average N50 of 80.4 kb, an average contig number of 9,084, and an average of 42% and 20% gene-coding and repetitive regions, respectively (Table 2) with LTR retrotransposons being the most common repeat type (Table 2; Supplementary Data Sheet 1). The genome metrics above were comparable to pan-genome datasets from the sister species *Zymoseptoria tritici*, which had an average core chromosome coverage of ~30X, an average N50 of 84.7 kb and average contig number of 1,088 (Feurtey et al., 2023). CATAStrophy (Hane et al., 2020) predictions for *S. pistaciarum* indicated non-haustorial hemibiotrophy (Table 3), which may correspond with prior reports of a 2 week latent phase (Crous et al., 2013). OcculterCut analysis of G:C content of the reference isolate assembly (Figure 2B) also indicated a bi-modal G:C distribution typical of hemibiotrophic fungi (Testa et al., 2016), with the AT-rich peak at approximately 38.6% G:C containing over a third (38.3%) of the genome length. Blastn vs. UNITE (Abarenkov et al., 2010) indicated >99.25% identity to the ITS region for all isolates to *Mycosphaerella pistaciarum*, however isolate 6 was also contaminated with plant DNA, and isolate 26 was contaminated with a Basidiomycete matching *Tomentella fuscograulosa* [NUC: UDB028526] (Supplementary Data Sheet 2). Despite varying levels of contamination in these two assemblies, the pan-genome survey approach of this study (Figures 2C,D) was capable of focusing on genome features relevant to the *S. pistaciarum* population as a whole, as genes specific only to isolates 6 and 26 were not the subject of further study. Assessment of the reference assembly via BUSCO indicated 98.7% completeness relative to the capnodiales_odb10 dataset (2020-08-05, genomes:13, BUSCOs:3578), with 3738/3786 BUSCOs detected completely (Supplementary Data Sheet 3).

**Table 2:** Summary of the repetitive DNA composition of *Septoria pistaciarum* reference isolate SPF8.

Repetitive DNA (isolate SPF8*)	
AT-rich regions (OcculterCut)	38.3% of genome peak: 38.6% G:C
Total Repetitive Regions (TE Tools)	15.3 Mb (35.41%)
Retroelements	23.26%
LINEs	6.88%
LTR elements	16.38%
Copia-like	2.84%
Gypsy-like	13.24%
DNA transposons	2.2%
Unclassified	8.92%
Low complexity/small RNA/simple repeats	1.03%

**Table 3:** Summary of pathogenicity gene features of *Septoria pistaciarum* reference isolate SPF8.

Predicted pathogenicity features (pan-genome)	
Total ortholog groups	21,174
Core: Conserved in all 27 isolates	7,743
Unique to a single isolate	7,079
Functionally-annotated (Pfam)	17,027
Secreted	3,032
Candidate pathogenicity effectors (Predector ≥2, cysteine ≥2)	59
Conserved in all 27 isolates	23

### 3.2 Local geographic distribution of the Greek *Septoria pistaciarum* pan-genome

Based on analysis of SNP-based distance trees the two regions—Pieria (north) and Larissa (central)—were distinct, whereas Fthiotida (south) was similar to both Pieria and Larissa regions (Figures 2A,C). While the geographic range and number of isolates sampled in this study is limited, based on the available data, we speculate that various events of long-distance dispersal (Golan and Pringle, 2017) caused by anthropogenic influences, spore dispersal, and infected nursery stock may be the cause of this clustering of isolates from the Fthiotida region with a relatively higher genetic divergence than their expected geographic distance. Such a clustering, where the populations were not strictly clustered based on their geographical origins, has also been reported in *Zymoseptoria tritici* populations (Mekonnen et al., 2020; Chedli et al., 2022). It may also indicate that these isolates belong to a population that might has experienced range expansions across the other two regions, which has resulted in admixture complemented by gene flow (Rogers and Rogers, 1999), gradually reducing the isolation by distance (Mills et al., 2007) of Fthiotida isolates. On the other hand, the extensive clustering into geographically-restricted lineages of isolates from Larissa and Pieria regions may indicate that these populations experienced a geographic isolation leading to local adaptation (Bazzicalupo, 2022). Future expansion of pan-genomic resources with a broader sampling to include surrounding regions or a globally-representative set of isolates of *S. pistaciarum,* might be able to confirm if isolates of the southern Fthiotida region represent a “wilder” population, relative those of the genetically narrower Pieria and Larissa regions. At present it is not clear whether pathways of movement through neighboring regions that were not sampled may have contributed to the transportation history of these isolates. Assemblies for all isolates possessed two versions of MAT1-1 type mating type genes, homologous to MAT-1-5/COX13 [ABS19615] and MAT1-1-6/APN2 [ABS19616] (Wilken et al., 2017; Supplementary Data Sheet 4). No MAT1-2 homolog was detected, suggesting limited potential for meiotic recombination across these three regions.

### 3.3 Potential secondary metabolite production

AntiSMASH prediction of secondary metabolite synthesis gene clusters (SMCs) in the reference isolate (SPF8) assembly, indicated homologs of 4 T1PKS (polyketide synthase) regions potentially encoding toxins similar to cercosporin (Chen et al., 2007), fusarubin/oxyjavanicin (Chowdhury et al., 2017), aspyridone A (Bergmann et al., 2007), fumonisin (Kamle et al., 2019), as well as melanin which may have a role in strengthening the pathogen cell wall (Jacobson, 2000). Also predicted were 15 non-ribosomal peptide synthase (NRPS)-like clusters potentially encoding the cytotoxic cyclic peptide serinocyclin (Krasnoff et al., 2007) and other unknown metabolites, and 5 terpene sythase clusters (Supplementary Data Sheet 5). Accurate prediction of secondary metabolite biosynthesis products remains challenging and requires further validation, however the clusters for serinocyclin and melanin were highly conserved. Overall, the predicted SMC profile of *S. pistaciarum* was similar to that previously reported for the relatively closely-related species *Zymoseptoria tritici* (Hassani et al., 2022), however *S. pistaciarum* had more NRPS SMCs whereas *Z. tritici* had more PKS SMCs (Supplementary Data Sheet 5).

### 3.4 Potential fungicide resistance

Although testing of differential fungicide efficacy and resistance across *S. pistaciarum* isolates was beyond the scope of this current study, it was possible to predict potential fungicide resistance mutations from pan-genomic data. Analysis of amino acid changes in the products of known fungicide resistance loci (Mair et al., 2016) revealed several potential resistance adaptations, inferred from reported resistance associated with equivalent mutations in other fungal species (Supplementary Data Sheet 6; Oliver et al., 2024). The CYP51A protein of all isolates contained amino acid residues corresponding to P216L, M220I, and H147Y of *Aspergillus fumigatus* CYP51A [NCBI: AF338659], which may confer azole resistance (Howard et al., 2009). Some isolates had mutations in CYP51B corresponding to deletions at Q287 and G412, and a mutation at S208T (relative to *Zymoseptoria tritici* [NCBI: AY253234]), that may be involved in DMI resistance (Stammler and Semar, 2011), CytB loci were not well represented across this pan-genome with data for only two isolates, however for these no intron/intein mutations were detected, and one deletion mutation corresponded to site D203 (relative to *Z. tritici* [NCBI: AY247413]), which in *Plasmopora viticola*, was involved in resistance to cyazofamid (Mounkoro et al., 2019). Beta-tubulin exhibited mutations corresponding to E198A and M257L (relative to *Aspergillus nidulans* [NCBI: M17519]), which may confer benzamidazole resistance (Leroux et al., 2002). A single isolate had a mutation in the OS1 protein corresponding to A350S of *Botrytis cinerea* [NCBI: AF435964] which may confer resistance to Fludioxonil (Ren et al., 2016). Overall, the mutations detected above and their corresponding resistance phenotypes inferred from related fungal species may reflect the broad application of boscalid + pyraclostrobin, fluxapyroxad, pyrimethanil, dodine and copper that was applied to all isolates in this study.

### 3.5 Prediction of pathogenicity effector genes

**Figure 3:**
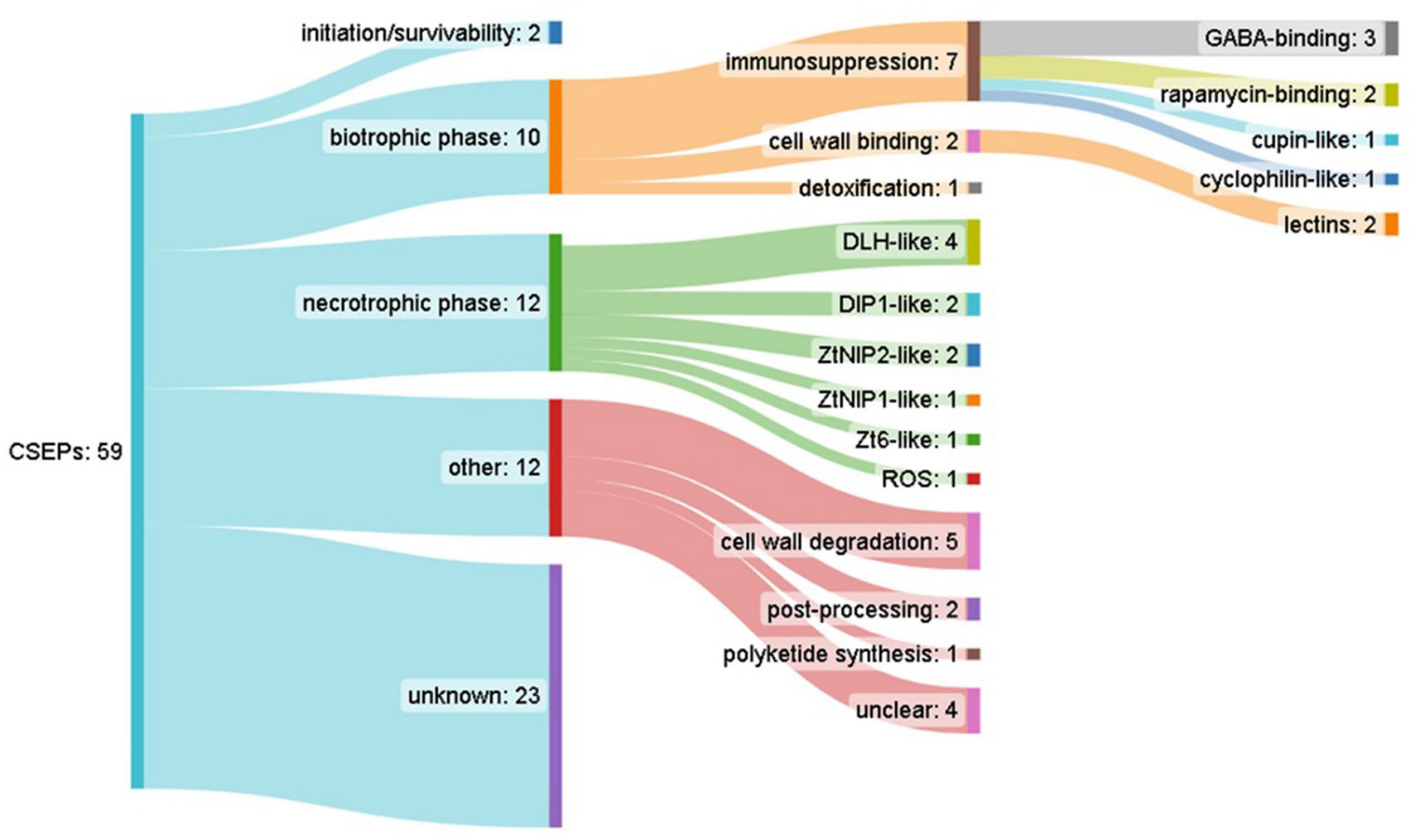
Summary of candidate secreted effector-like protein (CSEP) predictions across *Septoria pistaciarum* pan-genome-derived orthogroups, and their predicted functional annotations.

Comparative analysis of orthologous groups across the pan-genome indicated 21,174 ortholog groups (Supplementary Data Sheet 7), with isolates encoding an average of 11.2 K genes each, and 6,805 (32%) core groups containing a single member that was present in all isolates (Supplementary Data Sheet 8). Candidate secreted effector-like protein (CSEP) prediction across the representative pan-genome orthologous gene set, which in an effort to highlight high-priority effector candidates, was filtered for: predicted secretion (Predector—any method), Predector Score ≥ 2, and cysteine residues ≥ 2, which resulted in 59 CSEP-orthogroups (Figure 3) (Supplementary Data Sheet 8). Of these, notable CSEPs with conserved functional domains and/or with homology to confirmed effectors of other pathogen species (Supplementary Data Sheets 9, 10) are summarized below according to their speculative roles at various phases of *S. pistaciarum* infection. Overall, the predicted CSEP set indicated that *S. pistaciarum* may employ a combination of defensive effectors with roles in suppression of host defenses, and offensive effectors with a range of cytotoxic activities. Notably, some effector-like ortholog groups may have presented as divergent versions of the same protein (Table 4; Supplementary Data Sheet 8), suggesting region-specific adaptations may have occurred. There were 23/59 highly-conserved CSEP orthogroups, common to all 27 isolates, with another 5 similarly conserved sets of groups (WSC, PBP, DLH, ZtNip2 and Cutinase, Table 4) presenting as separate orthogroups that may be highly divergent versions of the same ortholog.

**Table 4:** Summary of candidate secreted effector-like protein (CSEP) predictions across *Septoria pistaciarum* pan-genome-derived orthogroups, and their predicted functional annotations.

Type/predicted role	Score	Ortholog Group	#prot/#isolates	FTH	LAR	PIE	Functional annotation	Len (aa)	#C
Unknown	3.712	SPIS_22831	27/27	9	9	9		172	6
Biotrophic-cell-wall-lectin-WSC	3.22	SPIS_07728	9/9	2	1	6	Pfam:PF01822(WSC)	112	9
Biotrophic-cell-wall-lectin-WSC	3.22	SPIS_26536	18/18	7	8	3	Pfam:PF01822(WSC)	112	9
Unknown	2.954	SPIS_02120	27/27	9	9	9		158	4
Necrotrophic-ROS	2.835	SPIS_12202	1/1	0	1	0	Pfam:PF00034(Cytochrom_C)	111	3
Other-post-processing	2.821	SPIS_22722	28/27	9	9	9	Pfam:PF01105(EMP24_GP25L)	213	2
Unknown	2.791	SPIS_03709	1/1	1	0	0		134	6
Necrotrophic-Zt6	2.789	SPIS_06740	27/27	9	9	9	Homology:Zt6, BghBEC1011, BgtAvrPm2, BghCSEP0055, BgtSvrPm3a1f1, BgAVRA13	117	4
Unknown	2.782	SPIS_25717	3/3	2	0	1		185	2
Unknown	2.746	SPIS_22513	27/27	9	9	9		216	4
Unknown	2.732	SPIS_02758	25/25	8	9	8		134	6
Unknown	2.732	SPIS_03496	1/1	0	0	1		134	6
Biotrophic-immunosuppression-GABA-binding-PBP	2.702	SPIS_00046	1/1	1	0	0	Pfam:PF01161(PBP); Localizer:nucleus	228	2
Biotrophic-immunosuppression-GABA-binding-PBP	2.702	SPIS_01458	6/6	4	2	0	Pfam:PF01161(PBP); Localizer:nucleus	228	2
Biotrophic-immunosuppression-GABA-binding-PBP	2.702	SPIS_02728	20/20	4	7	9	Pfam:PF01161(PBP); Localizer:nucleus	228	2
Other	2.698	SPIS_20847	27/27	9	9	9	Pfam:PF07452(CHRD)	236	4
Initial-spore-durability	2.641	SPIS_06676	1/1	1	0	0	Pfam:PF06766(Hydrophobin_2)	160	4
Biotrophic-immunosuppression-peptidylprolyl isomerase-rapamycin-binding	2.628	SPIS_11049	27/27	9	9	9	Pfam:PF00254(FKBP_C); Localizer:mitochondrion	179	4
Unknown	2.624	SPIS_10796	27/27	9	9	9		113	6
Unknown	2.598	SPIS_02801	4/4	0	2	2	Localizer:nucleus	199	2
Necrotrophic-DIP1	2.591	SPIS_23211	27/27	9	9	9	Homology:MoCDIP1; Localizer:chloroplast	349	7
Unknown	2.576	SPIS_06745	8/8	4	0	4	Localizer:chloroplast/mitochondrion	165	4
Unknown	2.558	SPIS_07982	15/15	3	8	4		185	2
Unknown	2.547	SPIS_26658	27/27	9	9	9		106	6
Unknown	2.514	SPIS_16527	1/1	0	0	1		89	3
Necrotrophic-DIP1	2.514	SPIS_23230	27/27	9	9	9	Homology:CfPDIP1	190	6
Unknown	2.508	SPIS_06094	25/25	7	9	9		187	4
Initial-spore-durability	2.44	SPIS_20995	27/27	9	9	9	Homology:PpCBEL	216	14
Necrotrophic-DLH	2.408	SPIS_01409	2/2	1	0	1	Pfam:PF01738(DLH)	287	2
Necrotrophic-DLH	2.408	SPIS_04361	3/3	0	0	3	Pfam:PF01738(DLH)	287	2
Necrotrophic-DLH	2.408	SPIS_04322	1/1	1	0	0	Pfam:PF01738(DLH)	287	2
Necrotrophic-DLH	2.408	SPIS_00267	21/21	7	9	5	Pfam:PF01738(DLH)	287	2
Unknown	2.38	SPIS_02605	9/9	4	1	4		185	2
Unknown	2.376	SPIS_25322	27/27	9	9	9	dbCAN:CE1; Localizer:chloroplast/mitochondrion	319	6
Biotrophic-immunosuppression-peptidylprolyl isomerase-rapamycin-binding	2.362	SPIS_18060	1/1	0	0	1	Pfam:PF00254(FKBP_C)	148	2
Other-PKS	2.361	SPIS_08900	8/1	0	0	1	Pfam:PF19373(DUF5948)	97	6
Other	2.343	SPIS_15222	1/1	0	0	1	Pfam:PF00565(SNase); Localizer:nucleus	245	8
Other-cell-wall	2.343	SPIS_15516	2/2	0	1	1	Pfam:PF06904(Extensin-like_C)	163	6
Other-cell-wall	2.343	SPIS_25738	27/27	9	9	9	Pfam:PF07745(Glyco_hydro_53); Localizer:mitochondrion	235	5
Unknown	2.334	SPIS_22743	27/27	9	9	9		229	3
Unknown	2.285	SPIS_05498	27/27	9	9	9		189	2
Necrotrophic-unknown	2.285	SPIS_24997	27/27	9	9	9	Homology:Foa4	107	8
Unknown	2.272	SPIS_24612	27/27	9	9	9		158	4
Other-post-processing	2.259	SPIS_26467	27/27	9	9	9	Pfam:PF01105(EMP24_GP25L)	212	2
Other	2.255	SPIS_04423	3/1	0	0	1	Pfam:PF00012(HSP70); Localizer:nucleus	200	2
Unknown	2.215	SPIS_25776	27/27	9	9	9		156	7
Biotrophic-immunosuppression-peptidylprolyl isomerase-cyclophilin	2.211	SPIS_03929	1/1	1	0	0	Pfam:PF00160(Pro_isomerase); Localizer:nucleus	323	2
Necrotrophic-ZtNIP2	2.199	SPIS_01666	21/21	6	6	9	Homology:ZtNIP2;	176	4
Necrotrophic-ZtNIP2	2.199	SPIS_01027	6/6	3	3	0	Homology:ZtNIP2;	176	4
Other-unknown	2.125	SPIS_12398	5/4	0	2	2	Pfam:PF00839(Cys_rich_FGFR)	71	4
Unknown	2.117	SPIS_25156	27/27	9	9	9		186	9
Biotrophic-detoxification-A-tomatine	2.113	SPIS_09627	2/1	0	0	1	Homology:CfTom1;	237	2
Unknown	2.11	SPIS_09528	3/1	0	0	1		189	8
Biotrophic-immunosuppressive-cupin-like	2.096	SPIS_22828	27/27	9	9	9	Pfam:PF00190(Cupin_1); Localizer:nucleus	269	7
unknown	2.079	SPIS_02601	1/1	1	0	0	Localizer:nucleus	100	2
Other-cell-wall	2.048	SPIS_03326	1/1	1	0	0	Pfam:PF01083(Cutinase);	228	9
Other-cell-wall	2.048	SPIS_05961	14/14	4	5	5	Pfam:PF01083(Cutinase);	228	8
Other-cell-wall	2.048	SPIS_27044	3/3	1	1	1	Pfam:PF01083(Cutinase);	228	8
Necrotrophic-ZtNIP1/HCE2-like	2.01	SPIS_22799	27/27	9	9	9	Homology:CfEcp2, ZtNIP1, UfRTP1, Pst18363;	181	5

#### 3.5.1 Spore survival prior to infection

Two CSEP orthogroups were categorized as having potential roles in spore durability and/or promoting initial colonization prior to infection. All 27 isolates possessed a PpCBEL lectin homolog (orthogroup SPIS_20995), which may promote colonization of leaf surface without directly causing virulence and may be recognized as a PAMP leading to HR (Gaulin et al., 2006). A single isolate (#4 of the FTH region) had a copy of orthogroup SPIS_03709, matching the Hydrophobin_2 domain. Other members of this family include the cerato-ulmins, a class of hydrophobins best studied in Dutch Elm Disease (Gallo et al., 2023). These CSEPs may promote infection rates over time by increasing spore resistance to dessication.

#### 3.5.2 Latent/biotrophic phase

There were 10 CSEP orthogroups which were categorized as having potential roles in the biotrophic/latent phase of infection, 7 of which appear to have roles in suppression of host defenses. Three orthogroups (SPIS_00046, SPIS_01458, SPIS_02728) matched to the PBP domain (periplasmic binding proteins), which may bind Gamma-aminobutyric acid (GABA). These 3 orthogroups are likely diverged from a common ortholog, as counts were distributed across the 27 isolates (1, 6, and 20 respectively), with SPIS_00046 specific to the FTH region, SPIS_01458 occurring in both FTH and LAR, and SPIS_02728 occurring across all 3 regions. The PBP-domain CSEPs could potentially interfere with GABA-regulated aspects of host metabolism and defense, as increased GABA concentration increases photosynthesis, reduces ROS production, regulates stomatal opening and increases biotic stress tolerance (Li et al., 2021). There were 2 CSEP orthogroups (SPIS_11049, SPIS_18060) which matched to the FKPBP_C domain, the former present in all 27 isolates, and the latter specific to a single isolate (#26—PIE). These are FK506-binding proteins—or FKBP-type peptidyl-prolyl cis-trans isomerases—which are functionally related to cyclophilins/immunophilins, and are receptors for ‘rapalog’-type immunosuppressant molecules including rapamycin, FK506, and cyclosporins. The best-studied example, rapamycin, has antifungal activity (Singh et al., 1979; Cruz et al., 1999), can increase tolerance to abiotic stresses (Dong et al., 2018), and can increase mitochondrial respiration and ROS production in host cells (Villa-Cuesta et al., 2014). Notably the CSEP SPIS_11049, common to all 27 isolates, was predicted by localizer to target the mitochondrion. Another CSEP SPIS_03929, only in isolate #3 of FTH, also matched to a cyclophilin-like domain with predicted nuclear localization. There were 2 very high-ranking (Predector score = 3.2) CSEP orthogroups (SPIS_07728, SPIS_26536) which matched the WSC domain, which are beta-glucan-binding lectins (Wawra et al., 2019) which have been reported to alter cell wall composition and suppress host PTI (Wawra et al., 2016). The two WSC-type CSEPs were another divergent set, with 9 and 18 isolates belonging to these groups respectively, with the former more prevalent in the Pieria region and the latter more prevalent in the FTH and LAR regions. There was also a cupin-like CSEP (SPIS_22828) common to all 27 isolates, localized to the nucleus, which may also have an immunosuppressive role (Yan et al., 2022).

#### 3.5.3 Necrotrophic phase

Aside from CSEP orthogroups that appear to support the biotrophic phase, there were several with putative necrotrophic effector functions. Four orthogroups (SPIS_00267, SPIS_01409, SPIS_04361, SPIS_04322) appear to be part of a larger divergent group (21, 2, 3, and 1 isolates, respectively) matching dienelactone hydrolases with the DLH domain. These may be involved in chlorocatechol degradation, may be required for virulence and potentially laterally-transferred between plant pathogen species (Gardiner et al., 2012). The less common orthogroups did not occur in the LAR region. Two CSEP groups (SPIS_23211, SPIS_23230) were both present in all 27 isolates, and matched (defense-inducing-protein) DIP1 pectate lyases, which can induce host HR, and increase hydrogen peroxide and alkanisation in host cells (Ashwin et al., 2018). There were several CSEP orthogroups which matched well-studied effectors of the closely-related wheat pathogen *Zymoseptoria tritici.*

A single orthogroup present in all 27 isolates (SPIS_22799) matched the necrosis-inducing effector ZtNIP1, and a divergent set of 2 orthogroups (SPIS_01666, SPIS_01027—present in 21 and 6 isolates respectively) matched the chlorosis-inducing effector ZtNIP2 (Ben M'Barek et al., 2015; Zhang et al., 2019). Another CSEP group (SPIS_06740) present in all 27 isolates matched the necrosis-inducing Zt6 ribonuclease, which may also have a dual-role in cytotoxicity against other microbes (Kettles et al., 2018). Other notable CSEP groups included a cytochrome C homolog (SPIS_12202) only present in 1 isolate (Sep_pis-14/LAR) and a Foa4 effector candidate homolog present in all isolates (SPIS_24997; Tintor et al., 2020).

#### 3.5.4 Other CSEPs with indeterminate roles

Among CSEP groups not assigned to the above categories, there were two groups (SPIS_25738 and SPIS_246467) which were present in all isolates, matching GH53 glycosyl hydrolases and EMP24/GP25L/P24/GOLD family proteins. The latter may have a role in transporting proteins from the endoplasmic reticulum in order to bind coat proteins to cytoplasmic domains, and effectors such PITC_013620 have this function (Li et al., 2022). There were also 3 groups matching CE5 Cutinase domains (SPIS_03326, SPIS_05961, SPIS_27044—from 1, 14 and 3 isolates respectively) which may have a role cell wall degradation during early infection.

## 4 Conclusion

We present these *S. pistaciarum* pan-genome resources as a foundational resource for pistachio disease surveillance and future effector gene discovery. Overall, these pan-genome-based analyses indicate that *S. pistaciarum* employs a combination of directly offensive effector proteins, as well as host-defense suppression during its relatively long biotrophic latent phase. A small set of toxic secondary metabolites and high-confidence effector candidate proteins have been generated, with indication of the relative conservation and/or regional-specificity of diverged sequence variants, which may help to direct focus to the study of the role in pathogenicity of these candidates in future validation studies. This study also demonstrates how previously under-studied patho-systems can now be rapidly surveyed using a combination of low-cost pan-genomic sequencing and the transfer of recent bioinformatic approaches and increasingly-informative pathogenicity-relevant datasets developed across other model fungal pathogen species.

## Data availability statement

The genome sequencing and transcriptome datasets generated for this study can be found in the NCBI database under BioProject: PRJNA1115914.

## Author contributions

AZ: Conceptualization, Data curation, Formal Analysis, Investigation, Software, Supervision, Validation, Writing – original draft, Writing – review & editing. AB: Investigation, Writing – original draft. NG: Data curation, Formal Analysis, Investigation, Methodology, Software, Supervision, Validation, Visualization, Writing – original draft, Writing – review & editing. MH: Data curation, Formal Analysis, Investigation, Methodology, Software, Supervision, Validation, Visualization, Writing – original draft, Writing – review & editing. MC: Investigation, Visualization, Writing – original draft, Writing – review & editing. DIT: Writing – original draft, Writing – review & editing. EP: Writing – original draft, Writing – review & editing. JH: Conceptualization, Data curation, Formal Analysis, Funding acquisition, Investigation, Methodology, Software, Supervision, Validation, Visualization, Writing – original draft, Writing – review & editing.

## Supplementary material

SUPPLEMENTARY DATA SHEET 1 Summary of genome assembly, gene and repetitive DNA metrics, for 27 genome assemblies of *Septoria pistaciarum*.

SUPPLEMENTARY DATA SHEET 2 Summary of contamination checks with BlastN versus UNITE, for 27 genome assemblies of *Septoria pistaciarum*.

SUPPLEMENTARY DATA SHEET 3 BUSCO gene completeness report versus the *Septoria pistaciarum* reference isolate SPF8 genome assembly.

SUPPLEMENTARY DATA SHEET 4 Screening results for mating type loci across 27 genome assemblies of *Septoria pistaciarum*.

SUPPLEMENTARY DATA SHEET 5 Secondary metabolite synthesis clusters predicted by AntiSMASH in the *Septoria pistaciarum* SPF8 reference isolate.

SUPPLEMENTARY DATA SHEET 6 Fungicide resistance mutations predicted by FRAST, for 27 genome assemblies of *Septoria pistaciarum*.

SUPPLEMENTARY DATA SHEET 7 Representative protein sequences for ortholog groups predicted in the *Septoria pistaciarum* pan-genome.

SUPPLEMENTARY DATA SHEET 8 Presence of ortholog groups across the 27 isolates of the *Septoria pistaciarum* pan-genome.

SUPPLEMENTARY DATA SHEET 9 Functional annotation and other pathogenicity metadata predicted by Predector, for pan-genome ortholog groups of *Septoria pistaciarum*.

SUPPLEMENTARY DATA SHEET 10 Protein feature locations of functional annotations predicted by Predector, for pan-genome ortholog groups of *Septoria pistaciarum*.
